# Cyclin-Dependent Kinase Five Mediates Activation of Lung Xanthine Oxidoreductase in Response to Hypoxia

**DOI:** 10.1371/journal.pone.0124189

**Published:** 2015-04-01

**Authors:** Bo S. Kim, Leonid Serebreni, Jonathan Fallica, Omar Hamdan, Lan Wang, Laura Johnston, Todd Kolb, Mahendra Damarla, Rachel Damico, Paul M. Hassoun

**Affiliations:** 1 Division of Pulmonary and Critical Care Medicine, Department of Medicine, Johns Hopkins University, Baltimore, MD 21224, United States of America; 2 Environmental Health Sciences, School of Public Health, Johns Hopkins University, Baltimore, MD 21224, United States of America; McGill University Department of Neurology and Neurosurgery, CANADA

## Abstract

**Background:**

Xanthine oxidoreductase (XOR) is involved in oxidative metabolism of purines and is a source of reactive oxygen species (ROS). As such, XOR has been implicated in oxidant-mediated injury in multiple cardiopulmonary diseases. XOR enzyme activity is regulated, in part, via a phosphorylation-dependent, post-translational mechanism, although the kinase(s) responsible for such hyperactivation are unknown.

**Methods and Results:**

Using an *in silico* approach, we identified a cyclin-dependent kinase 5 (CDK5) consensus motif adjacent to the XOR flavin adenine dinucleotide (FAD) binding domain. CDK5 is a proline-directed serine/threonine kinase historically linked to neural development and injury. We tested the hypothesis that CDK5 and its activators are mediators of hypoxia-induced hyperactivation of XOR in pulmonary microvascular endothelial cells (EC) and the intact murine lung. Using complementary molecular and pharmacologic approaches, we demonstrated that hypoxia significantly increased CDK5 activity in EC. This was coincident with increased expression of the CDK5 activators, cyclin-dependent kinase 5 activator 1 (CDK5r1 or p35/p25), and decreased expression of the CDK5 inhibitory peptide, p10. Expression of p35/p25 was necessary for XOR hyperactivation. Further, CDK5 physically associated with XOR and was necessary and sufficient for XOR phosphorylation and hyperactivation both *in vitro* and *in vivo*. XOR hyperactivation required the target threonine (T222) within the CDK5-consensus motif.

**Conclusions and Significance:**

These results indicate that p35/CDK5-mediated phosphorylation of T222 is required for hypoxia-induced XOR hyperactivation in the lung. Recognizing the contribution of XOR to oxidative injury in cardiopulmonary disease, these observations identify p35/CDK5 as novel regulators of XOR and potential modifiers of ROS-mediated injury.

## Introduction

Xanthine oxidoreductase (XOR) is a prototypical molybdo-flavoenzyme known for its role in purine catabolism, oxidizing hypoxanthine into xanthine and xanthine into uric acid [1]. XOR also generates reactive oxygen (ROS) and nitrogen (RNS) species. Specifically, XOR is a source of superoxide generated as a by-product of substrate oxidation. Thus, XOR can contribute to oxidative injury as part of the pathophysiology of numerous diseases including atherosclerosis, acute lung injury, and emphysema [2]. Expanding our understanding of how XOR is regulated is paramount to our knowledge of disease processes and search for effective treatments.

It has long been recognized that the enzymatic activity of XOR is dynamically regulated via transcriptional as well as post-translational mechanisms. Inflammatory cytokines, specifically tumor necrosis factor alpha, interleukin-1, and interferon gamma, or hypoxia hyperactivate XOR. This occurs independent of, or in excess of, de novo protein synthesis [3–5], implicating stimulus-dependent post-translational modification of the enzyme. *In vitro* data indicates that XOR is a phosphoprotein [5]. Hyperactivation of XOR by hypoxia is linked to the phosphorylation status of XOR. Pharmacologic studies have implicated multiple well-recognized kinases including p38 MAP kinase and ERK1/2 in the hyperactivation of the enzyme [6].

We have previously demonstrated hypoxia increases endothelial cell (EC) XOR activity via a post-translational modification, specifically a phosphorylation event involving p38 MAP kinase signaling [5]. We used Scansite 2.0 to identify other potential kinases [7] that could mediate XOR hyperactivation. We found threonine 222 on XOR as a potential target for cyclin-dependent kinase 5 (CDK5), an enzyme not previously linked to XOR or the regulation of ROS in EC.

CDK5 is a ubiquitously expressed serine/threonine protein kinase that has been implicated in a multitude of cellular processes including proliferation and differentiation as well as the regulation of cellular homeostasis [8]. While CDK5 is a member of the cyclin-dependent kinase family, the name is a misnomer in this case as CDK5 does not require cyclins for activity. Instead, CDK5 requires binding of its regulatory subunit p35 for activation [9]. The only characterization of CDK5 in the regulation of oxidative stress is in neurons where this kinase has been shown to phosphorylate and inactivate the hydrogen peroxide scavenger Prx2 [10]. Its role in regulating ROS generation via phosphorylation of XOR has not been previously described. In this manuscript, we characterize the role of CDK5 as an upstream regulator of XOR via phosphorylation of the threonine 222 site. Our results demonstrate that CDK5 and its activator p35 are necessary for hypoxia-induced XOR activation in pulmonary EC.

## Methods

All animal protocols were approved by the Johns Hopkins University Institutional Animal Care and Use Committee under protocol number MO11M438.

### Reagents and Cell culture

Primary adult rat lung microvascular endothelial cells (EC) were purchased from Cell Biologics Inc (Cell Biologics Inc., Chicago, IL) and maintained in basal medium supplemented with fetal bovine serum (FBS), epidermal growth factor, L-glutamine, and an antibiotic-antimycotic solution per manufacturer’s recommendations. Cells were analyzed between passage numbers 8 and 12 and for each experiment; within each experiment cells were matched by passage and lot numbers. 293A cell line was purchased from Life Technologies (Grand Island, NY) and was maintained according to manufacturer’s recommendations in media supplemented with 10% FBS (Corning Cellgro, Manassas, VA) and penicillin-streptomycin (Life Technologies, Grand Island, NY). Plasmids encoding hemagglutinin A (HA)-tagged CDK5 and the dominant negative CDK5 mutant (D144N) were purchased from Addgene (Cambridge, MA) [11]. On-Target plus siRNA targeting rat CDK5, p35 (CDK5r1), and non-targeting control siRNA were purchased from Dharmacon (Lafayette, CO, USA. Transfection of EC with duplex RNAs was carried out using Geneporter B reagent (Genelantis, San Diego, CA, USA) according to the manufacturer’s recommendations as detailed previously [12]. Lipofectamine 2000 reagent (Life technologies,Carlsbad, CA) was used for transfection of plasmid DNA per manufacturer’s recommendations.

### XOR expression

Dr. Mika Saksela (University of Helsinki, Finland) kindly provided the cDNA for human XOR (hXOR) (NM 000379.3). The hXOR-encoding insert was treated with Taq polymerase to generate 3’A-overhangs, then ligated to the donor vector, pcDNA 3.1/V5-His TOPO. After amplifying the donor vector-hXOR construct, it was incubated with an Echo vector in the presence of Cre recombinase, which recombined to form a fusion vector. The acceptor vector used for mammalian expression was a myc-poly histidine expression construct, pcDNA3.1mychisA purchased from Addgene (Cambridge, MA).

To generate the threonine to alanine substitution mutation, site directed mutagenesis was performed using the QuickChange lightning multi-site directed mutagenesis kit (Agilent Technologies, Columbia, MD) with the following primers: 5'-ctgaggctgaaagacgctcctcggaagcagc-3', 3'-gactccgactttctgcgaggagccttcgtcg-5'. Custom primers were purchased from Integrated DNA Technologies (Coralville, IA, USA). Plasmids encoding the wild type (T222) XOR (pcDNA3.1mychisAhXOR) or the alanine substitution (A222) were transiently transfected into 293A cells using Lipofectamine 2000, lysates were sonicated and run through a HisPur cobalt purification kit per the manufacturers’ protocol (Thermo Scientific, Waltham, MA). Purification elutions were subjected to SDS-PAGE and Western blotting to assess the presence of recombinant hXOR which was used for subsequent experiments.

### XOR enzymatic activity

Measurement of XOR activity was performed using a previously described fluorometric assay which utilizes the conversion of pterin into a fluorescent isoxanthopterin [13] with minor adjustments [6, 14]. Mouse lung tissue or EC cells were resuspended in a buffer containing 50mM KH_2_PO_4_, 10mM DTT, and 0.18mg/mL PMSF. Mouse lungs were homogenized in a bullet blender (Next Advance, Averill Park, NY), while cells were sonicated twice for 10 seconds on ice. Lysates were centrifuged at 13,000g for 15 minutes at 4°C and the supernatants were used in the assay immediately before measurement.

### CDK5 kinase activity

Kinase activity assay was performed as previously described [15]. Briefly, cell lysates and lung homogenates were pre-cleared with protein A/G agarose beads (Calbiochem, San Diego, CA) for 1 hour at 4°C. CDK5 was immunoprecipitated from 300μg of total pre-cleared protein samples overnight at 4°C using the anti-CDK5 polyclonal antibody (Santa Cruz, Santa Cruz, CA). Immune complexes were captured with protein A/G agarose beads, and washed three times with kinase activity buffer (Promega, Madison, WI). Beads were incubated with 10μg of histone H1 and magnesium/ATP cocktail (Promega, Madison, WI) at 30°C for 30 minutes. Reactions were terminated by addition of gel loading buffer and denaturation at 95°C for five minutes. 15μl of each reaction was subjected to Western blot analysis as previously mentioned, and phosphorylated histone H1 was detected using a polyclonal S139 antibody (R&D systems, Minneapolis, MI). In addition, total H1 was detected using a monoclonal antibody (R&D systems, Minneapolis, MI). Assay was quantified by densitometry analysis.

### Immunoprecipitation and protein purification

XOR and CDK5 were precipitated from 300μg of protein lysates pre-cleared with 25μl of protein A/G agarose beads (50% slurry) at 4°C for 3 hours. Proteins were immunoprecipitated by addition of the respective primary antibody, anti-CDK5 (Santa Cruz, Santa Cruz, CA), anti-XOR (Santa Cruz, Santa Cruz, CA,), or a species control IgG and incubated overnight at 4°C. Immune complexes were captured with protein A/G agarose beads slurry and washed three times in lysis buffer or kinase activity buffer. One-fifth of the immunoprecipitation reaction was subjected to Western blot analysis, or for CDK5 activity measurements.

### *In vitro* phosphorylation assay

Human myc-his-tagged XOR (pcDNA3.1mychisAhXOR) was expressed in 293A cells and purified using the HisPur cobalt purification kit (Thermo Scientific 90090) according to the manufacturer’s recommendations. Equal amounts of purified XOR protein was added to each reaction containing immune-precipitated CDK5, 1x kinase assay buffer, and Mg^2+^/ATP cocktail. Reactions were incubated at 30°C for 20 minutes and termination by addition of gel loading buffer and incubation at 95°C for 5 minutes. Each reaction was resolved on SDS-PAGE and probed with anti-phospho-threonine antibody (Cell Signaling, Boston, MA) and with an anti-myc antibody (Cell Signaling, Boston, MA).

### Western blot analysis

Cell lysates were generated by homogenization in Cell Lysis Buffer (Cell Signaling, Boston, MA) supplemented with 1x protease inhibitor cocktail (Sigma Chemical Co., St. Louis, MO, P8340) and sonicated, twice using a Fischer Scientific sonicator FB50, then cleared by centrifugation. 40μg of protein per sample were resolved by electrophoresis on 12% tris-glycine gels from Life Technologies (Carlsbad, CA) and transferred onto a polyvinylidene difluoride membrane (Bio-Rad, Richmond, CA). Specific proteins were detected with the following antibodies, anti-XOR (Santa Cruz, Santa Cruz, CA, #sc-22006), anti-GAPDH-HRP (Cell Signaling, Boston, MA, #3683), anti-Cdk5 (Santa Cruz, Santa Cruz, CA, #sc-6247), anti-p35 C-19 (Santa Cruz, Santa Cruz, CA, #sc-820), anti-p35 N-20 (Santa Cruz, Santa Cruz, CA, #sc-821), anti-HA tag (Santa Cruz, Santa Cruz, CA, #sc-57592), anti-histone (Cell Signaling, Boston, MA, #2935), anti-phospho-H1 (Cell Signaling, Boston, MA, #2577), anti-myc antibody (Cell Signaling, Boston, MA, #2272), and anti-phospho-threonine (Cell Signaling, Boston, MA, #9391) where indicated. Immune complexes were detected using chemiluminescence (ECL; Amersham Pharmacia Biotech, Piscataway, NJ, USA).

### Hypoxia exposure

Cultures treated with the appropriate conditions were placed in humidified airtight incubation chambers (Billups-Rothenberg, Del Mar, CA) which were gassed with 1% O_2_, 5% CO_2_, and balance N_2_. For the duration of the specified hypoxia exposures the incubation chambers were kept inside a 37°C incubator. Normoxic cells were kept in a tissue culture incubator maintained at 5% CO_2_ and 37°C.

Male C57/bl6 mice at 8 weeks of age (n = 5) were randomized to normoxic or hypoxic exposure. Hypoxic mice were exposed to 10% O_2_ in a plexiglass chamber (Biospherix, Inc.) monitored by Pro:Ox oxygen controller (model 350; Reming Bioinstruments, Redfield, NY), as previously described [16]. Designated groups were administered with 3mg/kg olomoucine in 1% DMSO or vehicle via an intraperitoneal injection 24 hours before exposure to hypoxia, and another dose 4 hours prior to hypoxia exposure.

### Statistical analysis

Data are shown as the mean and standard error. Unpaired or paired Student *t*-test as well as ANOVA were used for statistical comparisons when appropriate. A *P* value of < 0.05 was considered significant. Data were analyzed using GraphPad Prism 5.

## Results

### Hypoxia-induced hyperactivation of XOR is dependent on a phosphorylation event

**Fig 1:**
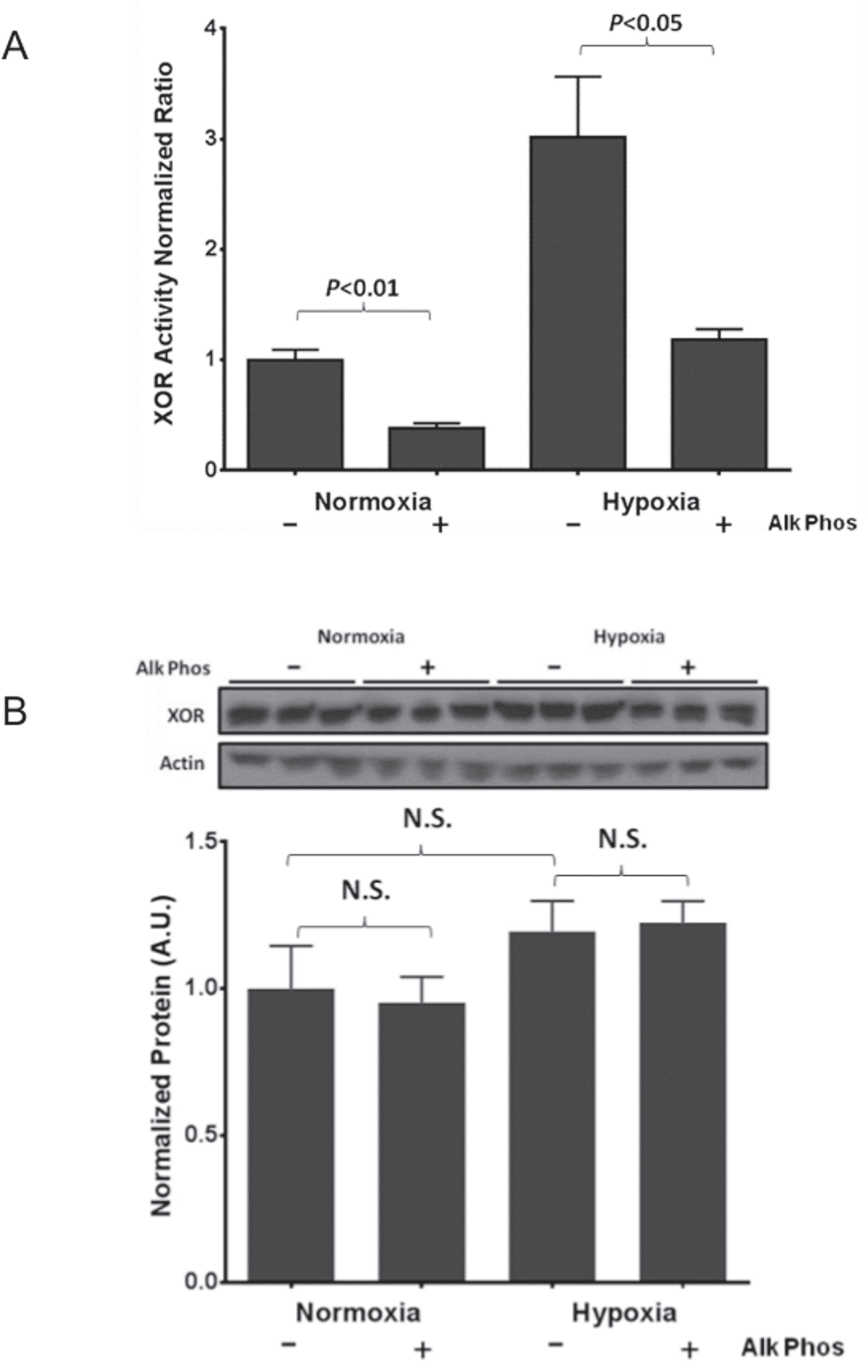
Hypoxia-induced hyperactivation of XOR is dependent on a phosphorylation event. XOR activity was measured in lysates from EC exposed to hypoxia (1% O_2_, 5% CO_2_ and balance nitrogen) or normoxia (21% oxygen, 5% CO_2_, and balance nitrogen) for 24 hours followed by treatment with vehicle (DMSO) or alkaline phosphatase (Alk Phos). XOR activity in both normoxic and hypoxic conditions is significantly reduced in cell extracts treated with Alk Phos (A). n = 3–5 for each condition. This hypoxia-induced increase in XOR activity is independent of changes in protein expression as demonstrated by Western blot and densitometric analysis (B). n = 3 for each group.

We have previously demonstrated that hypoxia-mediated upregulation of XOR activity is partially dependent on a post-translational mechanism, such as a phosphorylation event [5]. Here we demonstrate a significant increase in XOR activity in EC exposed to hypoxia (1% O_2_ for 24 hours) compared to normoxia (21% O_2_), which was abrogated by treatment with alkaline phosphatase (Alk phos) (Fig 1A). Interestingly, Alk phos treatment inhibited XOR activity at normoxic conditions demonstrating the dependence of XOR activity on phosphorylation even at baseline. Hypoxia-induced XOR activation was independent of changes in protein expression as demonstrated by Western blot and densitometric analysis (Fig 1B), again consistent with a post-translational mechanism of activation. Western blotting of XOR protein under these conditions demonstrating no significant differences in protein levels suggested that the loss of XOR activity was not secondary to non-specific Alk phos mediated degradation of XOR (Fig 1B).

**Fig 2:**
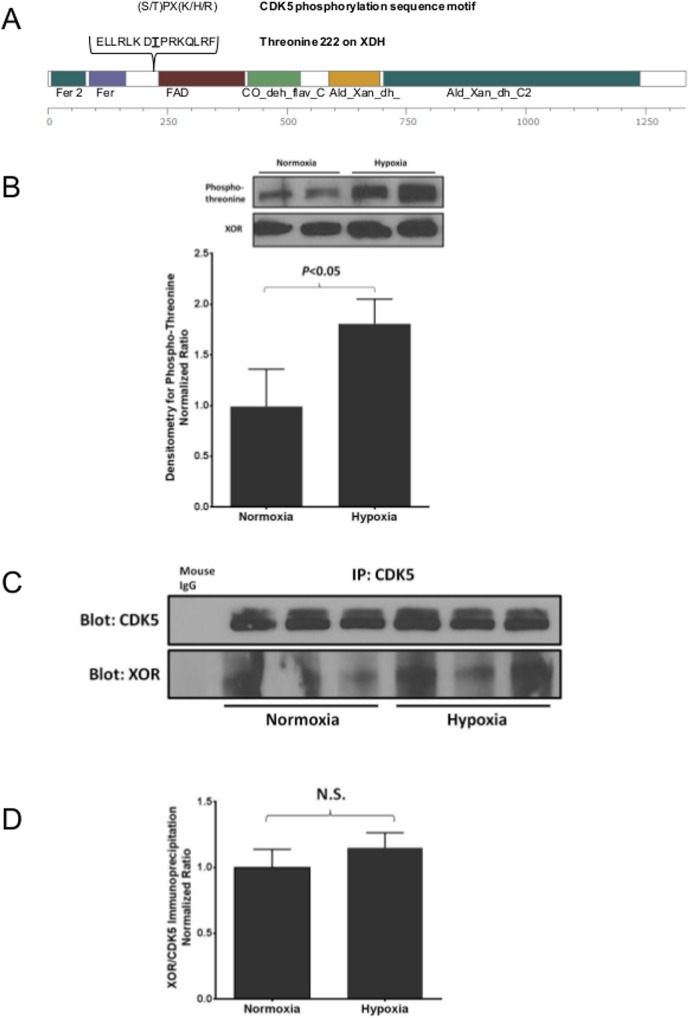
CDK5 is associated with XOR under normoxic and hypoxic conditions. Using *in silico* analysis, we identified a CDK5 consensus motif adjacent to the flavin adenine dinucleotide (FAD) binding domain (A). There was increased XOR phospho-threonine expression in cells exposed to hypoxia compared to normoxia as determined by Western blot analysis (B). n = 4 for each group. CDK5 immunoprecipitated from EC lysates after exposure to hypoxia or normoxia was subjected to SDS-PAGE and probed for XOR and mouse IgG control. Western blot analysis revealed an association in a complex of CDK5 and XOR in both hypoxic and normoxic conditions (C). Densitometric analysis demonstrated no change in XOR/CDK5 protein ratios comparing normoxic and hypoxic conditions (D). n = 3–5 for each condition.

Using Scansite 2.0 to find potential kinase target sites in the XOR amino acid sequence, we observed that threonine 222 was part of a CDK5 consensus sequence (Fig 2A) [7]. To determine if activation was associated with changes in the phosphothreonine content of XOR, we evaluated the phosphothreonine reactivity of XOR protein immunoprecipitated from EC exposed to normoxia and hypoxia for 24 hours. After hypoxia, XOR had a significant increase in phosphothreonine as demonstrated by Western blot and densitometry (Fig 2B). Based on these findings we sought to find a physical interaction between the serine/threonine kinase CDK5 and XOR; therefore, we co-immunoprecipitated the two endogenous proteins from EC. After exposure to hypoxia (1% O_2_ for 24 hours) or normoxia (21% O_2_), cellular protein was immunoprecipitated with anti-CDK5 antibodies or control immunoglobulin and Western blot analysis was performed. As shown in Fig 2C, CDK5 and XOR are associated in a protein complex under both hypoxic and normoxic conditions. There were no changes in the respective protein levels between the two exposures as demonstrated by densitometric analysis (Fig 2D).

**Fig 3:**
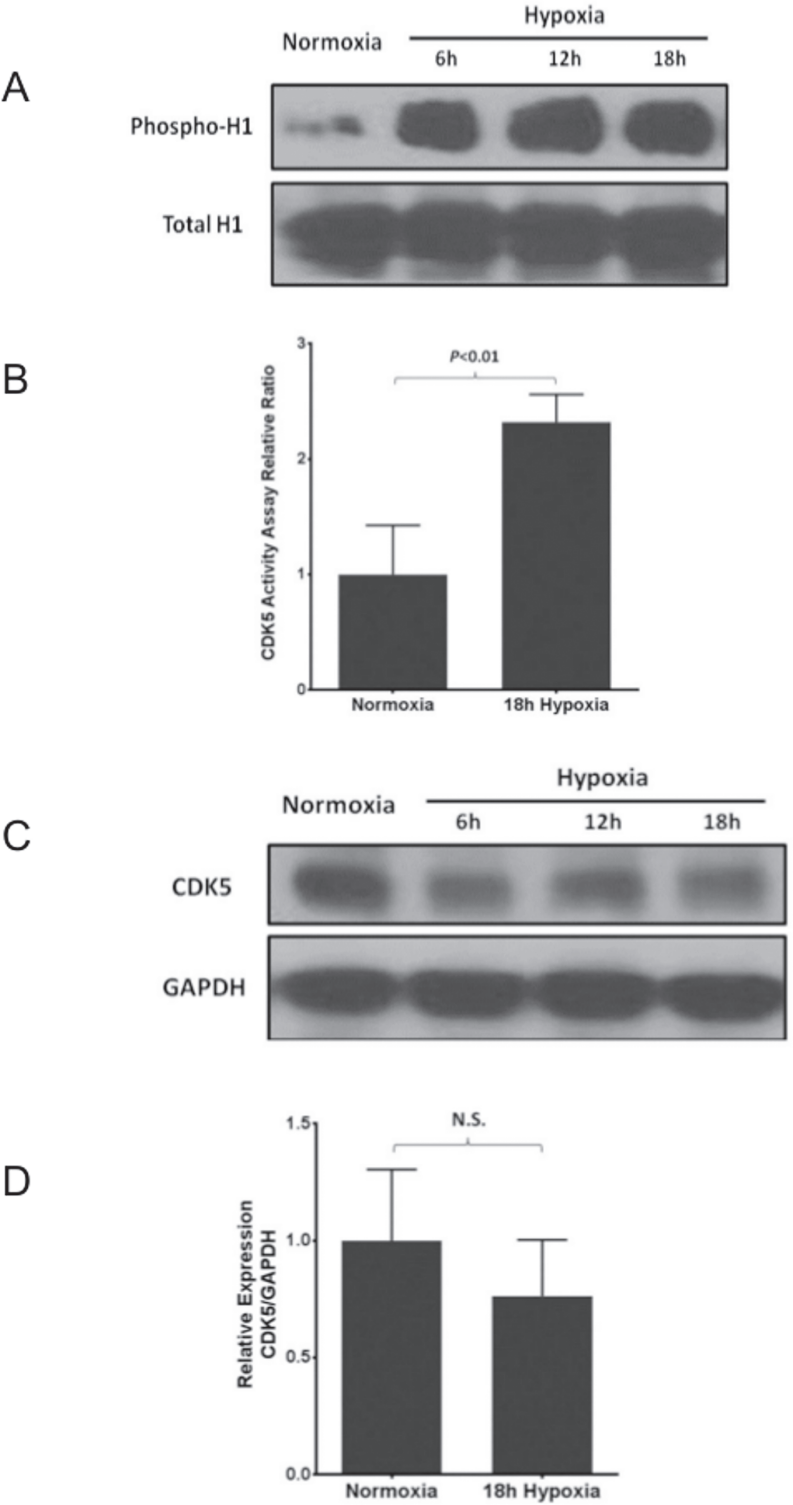
CDK5 activity is upregulated in response to hypoxia. CDK5 was immunoprecipitated from EC after exposure to hypoxia (1% O_2_, 5% CO_2_ and balance nitrogen) at durations of 6, 12, and 18 hours and incubated with histone H1. Reactions were subjected to SDS-PAGE and probed for phospho-histone H1 as an indirect measure of CDK5 kinase activity. Western blot analysis showed the levels of phospho-histone H1 compared with total H1 levels increased significantly in a hypoxia exposure duration dependent manner compared to normoxia (A). Densitometric analysis demonstrated significant increase in CDK5 activity comparing normoxia versus hypoxia for 18 hours duration (B). The protein levels of CDK5 did not change significantly for all tested time points (6, 12, and 18 hours) comparing normoxic and hypoxic conditions as demonstrated by Western blot (C) and densitometric analysis of the 18 hour time point (D). n = 4 for each group.

To further test whether CDK5 activity was functionally different between complexes from normoxic and hypoxic cells, we analyzed kinase activity of the endogenous protein. CDK5 was immunoprecipitated from normoxia and hypoxia exposed cell lysates and added to a mixture of histone H1, Mg^2+^, ATP and buffer. H1 phosphorylation, a CDK5 target, was assessed by Western blotting. The phosphorylation status of H1 was thus a marker of CDK5 activity. There was a significant and time-dependent increase in CDK5 activity in hypoxic compared to normoxic EC (Fig 3A). Densitometric analysis demonstrated a significant increase in CDK5 activity at 6, 12, and 18 hours of hypoxia exposure (Fig 3B, 18 hour time point). Total histone H1 and CDK5 protein levels did not change significantly in hypoxia compared to normoxia as demonstrated by Western blot (Fig 3A and 3C, respectively) and densitometry (Fig 3D), indicating changes in CDK5 activity are independent of changes in protein. Thus, we evaluated changes in known regulators of CDK5 activity.

**Fig 4:**
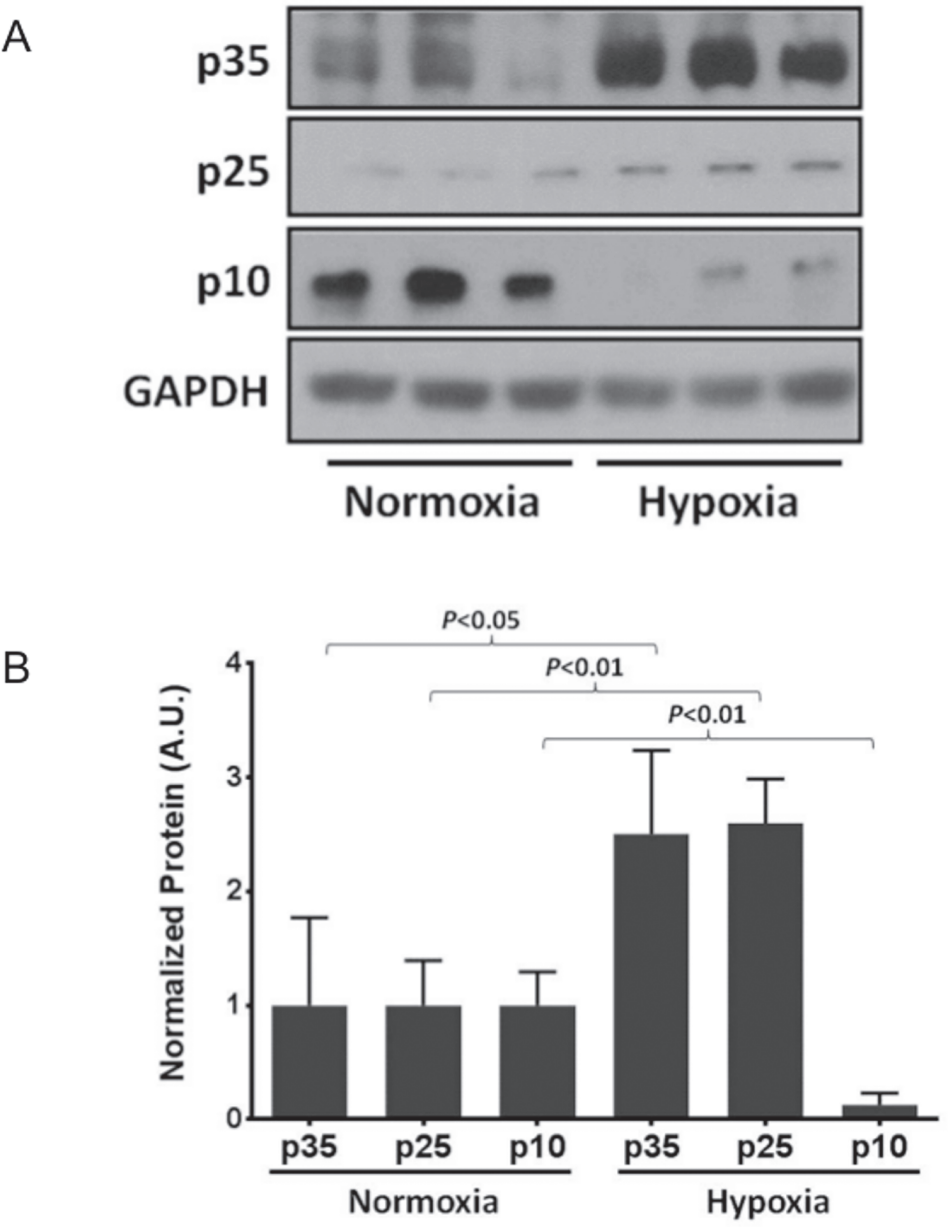
Hypoxia upregulates protein levels of the regulatory subunits p35 and p25 but not CDK5. Hypoxia augments CDK5 activity without significant increase in protein expression. Cell lysates from EC exposed to hypoxia (1% O_2,_ 5% CO_2_ and balance nitrogen) compared to normoxia for 18 hours have increased protein levels of the CDK5 regulatory subunits p35 and p25 as demonstrated by Western blot (A) and densitometric analysis (B). n = 3–5 for each group. Also shown is a decreased protein level of the cleaved fragment p10, a known inhibitor of CDK5 activity (A and B).

Interestingly, there were significant increases in the protein levels of p35 and p25, the regulatory activating subunits of CDK5, in EC exposed to hypoxia (Fig 4A and 4B). In contrast, the protein fragment p10, a known inhibitor of CDK5 kinase activity, was significantly downregulated by exposure to hypoxia as compared to normoxia. Taken together, these experiments demonstrate CDK5 and XOR form a protein complex in EC in hypoxia and normoxia without relative change in protein expression between the two conditions; however, hypoxia causes an increase in CDK5 kinase activity concomitant with an upregulation of the activating subunits p35 and p25 and downregulation of the inhibitor p10.

### CDK5 and p35 are necessary for hypoxia-induced hyperactivation of XOR in EC

**Fig 5:**
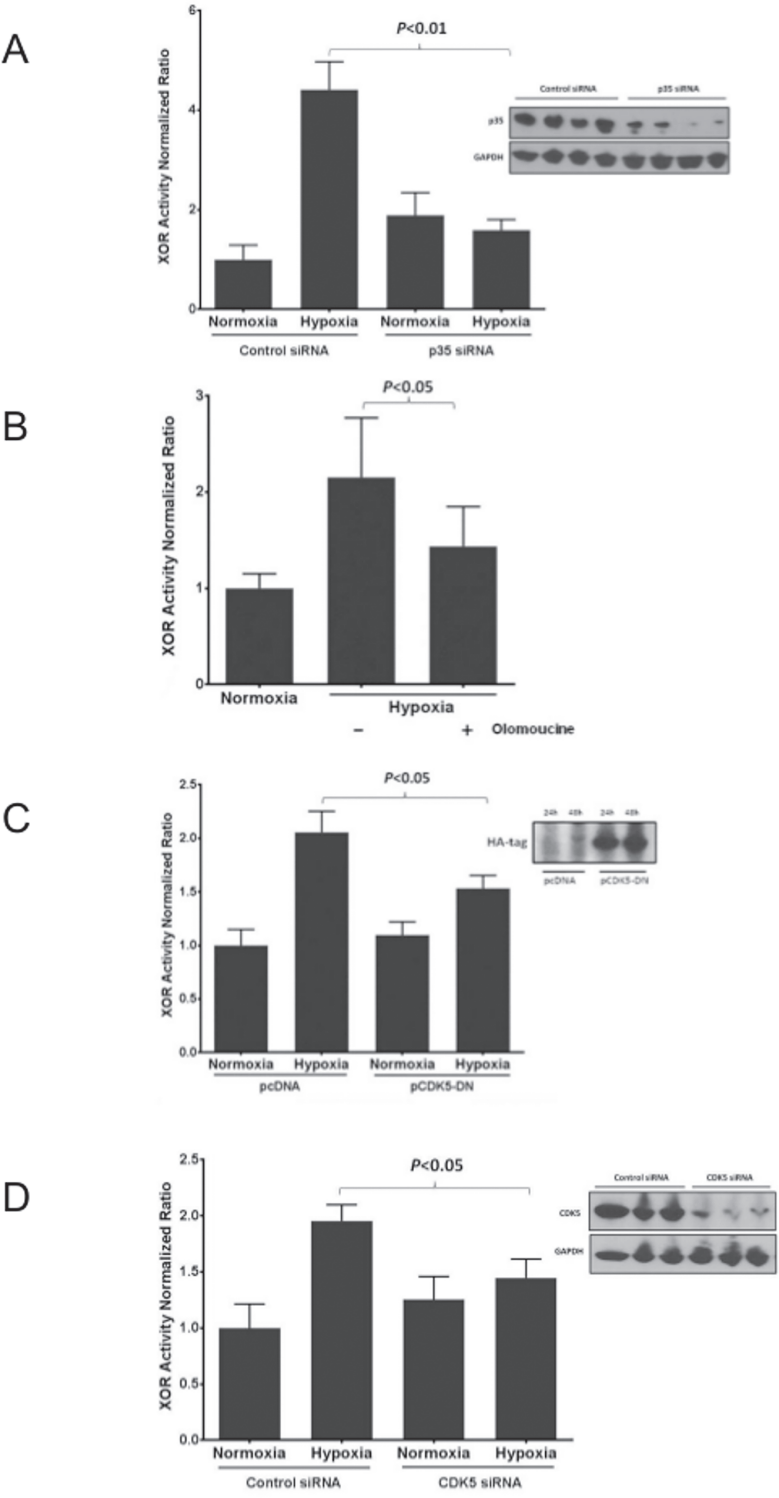
CDK5 and its regulatory subunit p35 are necessary for hypoxia induced hyperactivation of XOR activity. Using siRNA directed against p35/CDK5r1, we demonstrated effective knockdown of p35 by Western blot (inset) compared to the negative control siRNA. XOR activity measured in EC transfected with p35/CDK5r1 siRNA and exposed to hypoxia (1% O_2_, 5% CO_2_ and balance nitrogen) for 24 hours revealed effective suppression of the upregulation of XOR activity (A). n = 3–5 for each group. In parallel, cell lysates from EC exposed to hypoxia (1% O_2_, 5% CO_2_ and balance nitrogen) or normoxia for 24 hours in the presence of the CDK5 inhibitor olomoucine (100 μM) or vehicle (DMSO) were measured for XOR activity as described in Methods. XOR activity is significantly elevated after exposure to hypoxia in the cells pretreated with vehicle (DMSO), which is abrogated by olomoucine pretreatment (B). n = 6 for each condition. In parallel experiments, EC were transfected with a plasmid overexpressing the kinase inactive mutant CDK5 (D144N) or an empty vector prior to exposure to hypoxia or normoxia for 24 hours. Effective transfection was demonstrated by Western blot probing for the HA-tag (inset). XOR activity was significantly increased in EC transfected with an empty vector and exposed to hypoxia compared to the dominant negative CDK5 construct (C). n = 3–5 for each group. CDK5 was effectively knocked down in EC with the specific CDK5 siRNA (inset). CDK5 siRNA significantly prevented the upregulation of XOR activity after exposure to hypoxia (1% O_2_, 5% CO_2_ and balance nitrogen) for 24 hours compared to negative control siRNA (D). n = 3–5 for each group.

Previous studies have demonstrated that, in cultures of neurons, CDK5 becomes activated when bound to its regulatory subunit p35 and hyperactivated when bound to the cleavage product p25 [17]. We examined the role of p35 in hypoxia-induced XOR hyperactivation in EC using siRNA specific for p35/CDK5r1 (Fig 5 inset). Treatment with p35/CDK5r1 specific siRNA significantly prevented the hypoxia-induced increase in XOR activity observed in EC transfected with the negative control siRNA (Fig 5A). To further characterize the role of CDK5 in hypoxia-induced XOR hyperactivation, we used the known pharmacological inhibitor olomoucine at a 100μM concentration. As shown in Fig 5B, there was significant attenuation of the hypoxia-induced XOR activity in cells pre-treated with olomoucine (Fig 5B). In parallel experiments, we transfected EC prior to hypoxia exposure with a plasmid overexpressing the kinase inactive mutant CDK5 (D144N), which functions as a dominant negative kinase [11]. Cells transfected with CDK5 (D144N) had a significantly blunted XOR activity in response to hypoxia exposure compared to EC transfected with the wildtype (WT) CDK5 (Fig 5C). Similarly, cells transfected with CDK5 siRNA had an abrogated hypoxia-induced XOR activation compared with cells transfected with the negative control siRNA (Fig 5D). Taken together, our findings establish the necessity for an intact CDK5 with kinase activity, most likely regulated by its binding partner p35, for hypoxia-induced XOR hyperactivation in EC.

### CDK5 is sufficient to hyperactivate XOR activity

**Fig 6:**
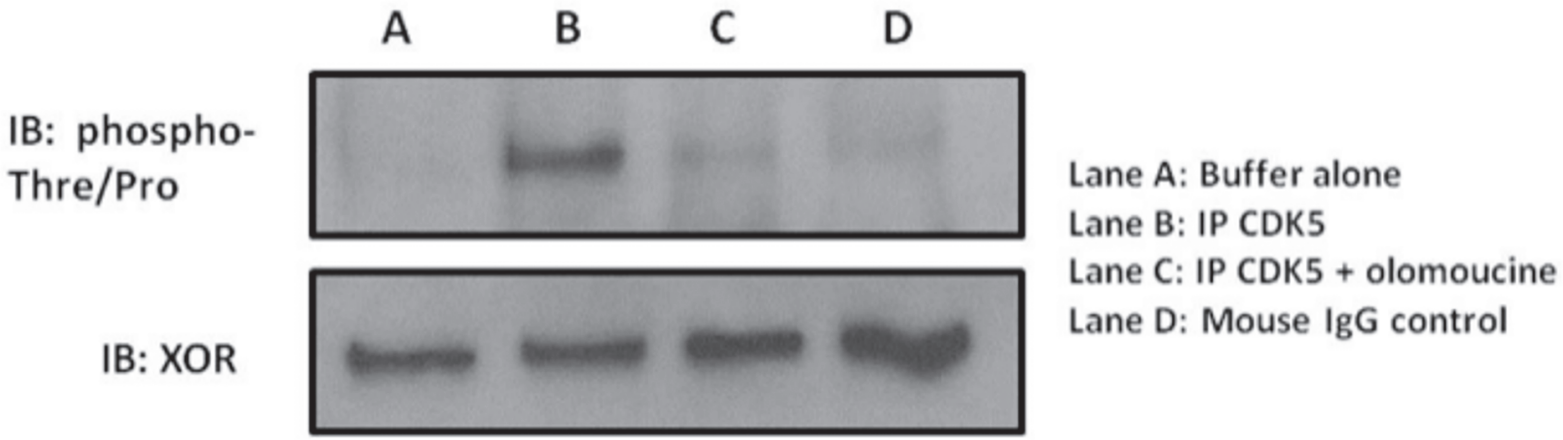
CDK5 is sufficient to phosphorylate XOR in a cell free reaction. Human poly-histidine tagged XOR expressed in 293A cells and purified via chromatography was incubated with buffer alone, immunoprecipitated CDK5 with or without olomoucine, and with mouse IgG as a negative control along with Mg^2+^/ATP. Each reaction was resolved on SDS-PAGE and probed with anti-phospho-threonine-proline antibody and anti-XOR antibody. CDK5 was sufficient to phosphorylate XOR at a phospho-threonine-proline site (lane B). The amount of XOR protein in each reaction was uniform as shown on the immunoblot.

Once we determined CDK5 was necessary for hypoxia-induced hyperactivation of XOR, we tested whether CDK5 was sufficient to phosphorylate XOR and induce increased enzymatic activity in a cell free system. We generated poly-histidine tagged human XOR (hXOR) protein in 293A cells via transient transfection and purified it using chromatography as detailed in Methods. The hXOR was then incubated in the absence or presence of immunoprecipitated CDK5 with and without its inhibitor olomoucine with ATP and Mg^2+^. Each reaction was resolved on SDS-PAGE and probed with anti-phospho-threonine/proline antibody, demonstrating the presence of CDK5 is sufficient to significantly increase hXOR phosphorylation at a threonine and/or proline site (Fig 6).

**Fig 7:**
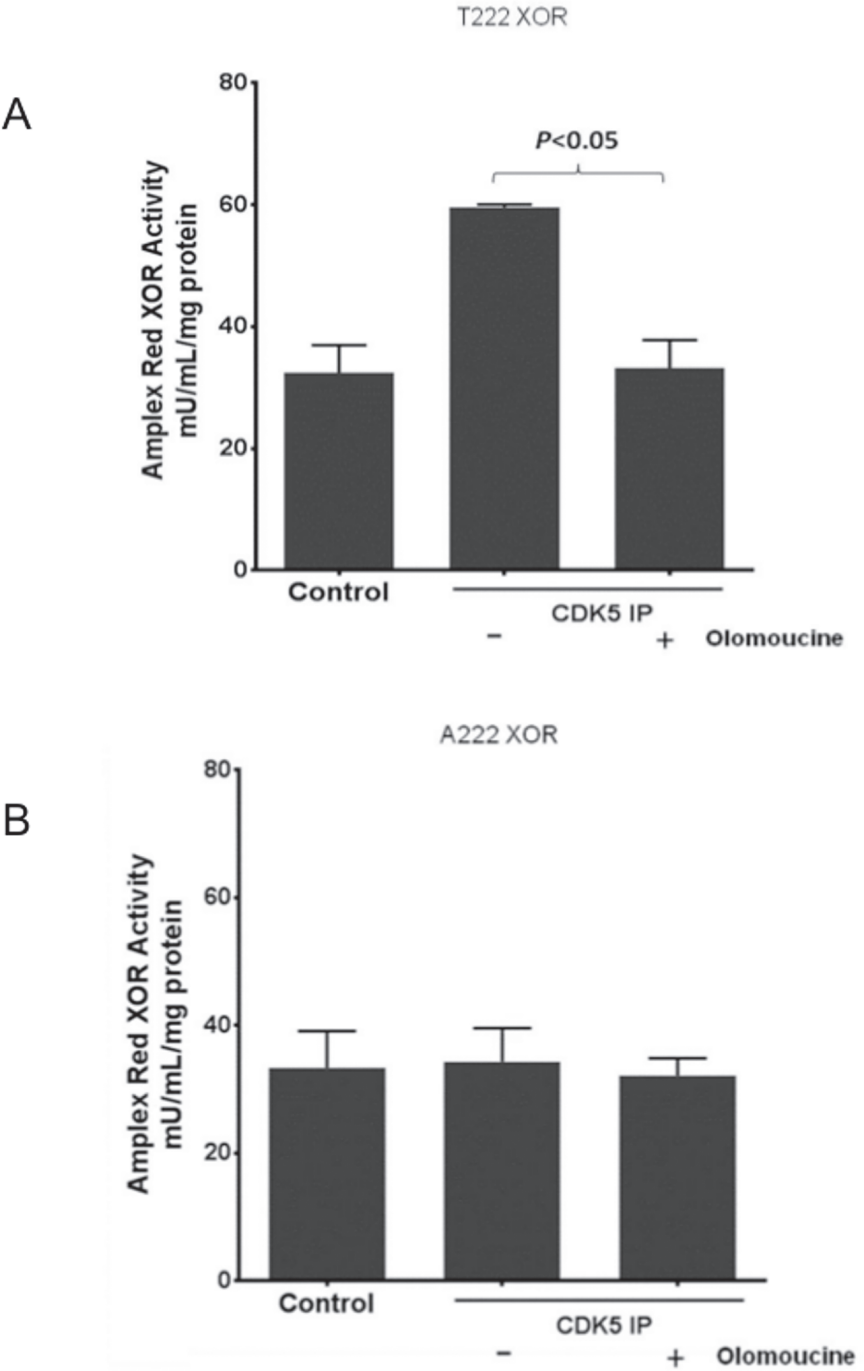
CDK5 is sufficient to upregulate XOR activity in a cell free reaction. Recombinant hXOR proteins (wild type T222 versus alanine mutant A222) were incubated in the presence or absence of immunoprecipitated CDK5 with Mg^2+^/ATP and XOR activity was measured using an Amplex red assay. The activity of T222 XOR was increased in the presence of CDK5 and abrogated by olomoucine treatment (A). n = 3–5 for each group. There was no change in XOR activity with the A222 mutant in the presence of CDK5 (B).

In parallel, we generated expression constructs encoding a poly-histidine-tagged hXOR with an intact threonine at residue 222 (T222) of the CDK5 consensus sequence and an alanine mutant (A222) that cannot be phosphorylated at residue 222. Both recombinant hXOR proteins were then incubated in the presence or absence of immunopurified CDK5 with ATP and Mg^2+^. The XOR enzymatic activity was assessed using the Amplex Red assay in the presence or absence of olomoucine. There was no demonstrable difference in the activity of T222 versus A222 XOR at baseline indicating that T222 was not obligatory for basal enzymatic activity. The activity of T222 XOR was significantly increased in the presence of CDK5 (Fig 7A), coincident with increased phosphorylation of XOR (Fig 6). In contrast, activity of the A222 XOR protein was insensitive to CDK5 (Fig 7B). CDK5 enzymatic activity was required for the induction of XOR and was inhibited by olomoucine treatment, demonstrating specificity for the kinase. These results demonstrate that CDK5 is not only necessary but sufficient for the hyperactivation of XOR activity through XOR phosphorylation of threonine 222.

### CDK5 is necessary for hypoxia-induced hyperactivation of XOR *in vivo*

**Fig 8:**
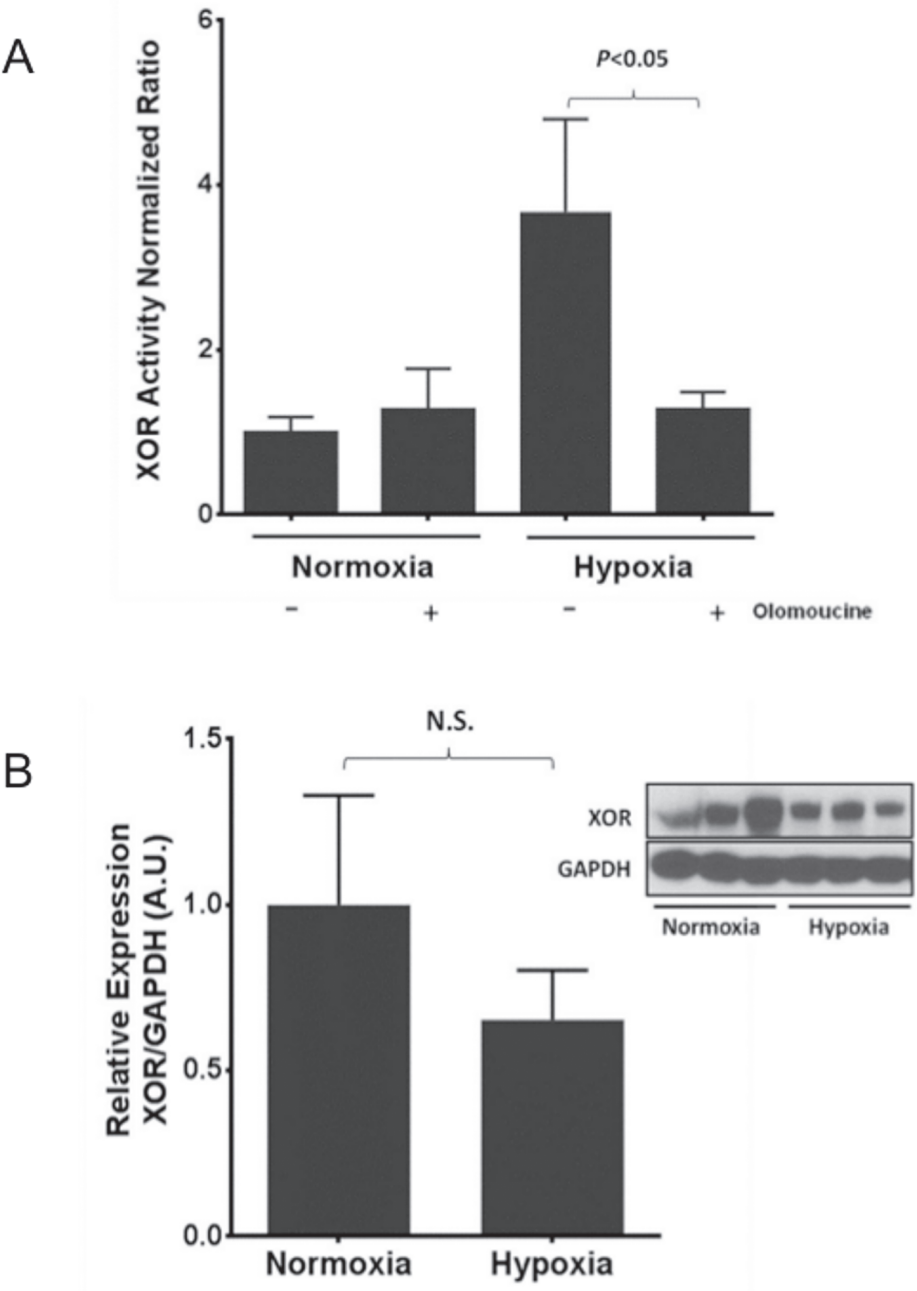
CDK5 is necessary for hypoxia-induced hyperactivation of XOR in mouse lung. C57/Bl6 mice were exposed to hypoxia (10% O_2_) or room air for 24 hours with designated groups receiving pretreatment with olomoucine (3mg/kg) versus vehicle. Lungs were harvested and homogenized for XOR activity. There was significant hypoxia-induced XOR activation which was dependent on CDK5 activity (A). n = 5 for each group. XOR activation was not dependent on increased protein levels as demonstrated by Western blot and densitometric analysis (B).

We have shown that CDK5 is necessary for the hypoxia-induced hyperactivation of XOR in EC. To further test this interaction *in vivo*, we exposed 8 week old male C57/Bl6 mice to hypoxia (10% O_2_) or room air for 24 hours with designated arms receiving pretreatment with olomoucine (3mg/kg) versus vehicle delivered via peritoneal injection. At the end of exposure to hypoxia, there was significant lung XOR hyperactivation compared to normoxia (Fig 8A), which was completely abrogated by pre-treatment with olomoucine, demonstrating *in vivo* that hypoxia-induced hyperactivation of XOR is CDK5 dependent. This hyperactivation of XOR was not dependent on increased protein levels as demonstrated by Western blot and densitometric analysis (Fig 8B).

## Discussion

The role of XOR in the pathophysiology of disease has been widely investigated and the enzyme’s importance has been substantiated by studies in a multitude of pathologies including cardiovascular disease [18, 19], ischemia-reperfusion injury [20], acute lung injury [6], and emphysema [14, 21–23], all of which are syndromes characterized by tissue hypoxia, a known major activator of XOR. Our aim in this study was to identify potential upstream mediators of XOR activation thereby gaining insight into the mechanisms of XOR regulation in the context of hypoxia. In our previous work, we identified p38 and casein kinase 2 (CK2) as potential regulators of hypoxia-induced XOR hyperactivation [5]; however, the CK2 inhibitor used in the study was non-specific and had off-target effects. Furthermore, we demonstrated that simple activation of p38 by sorbitol or arsenite, known stimulators of p38, did not significantly induce the phosphorylation of XOR thus suggesting that p38 alone was not sufficient to activate XOR. Although there are many potential kinases and regulators of XOR activity, in the present study we identified a specific CDK5 phosphorylation site in the XOR amino acid sequence at threonine 222 and established a critical role for CDK5 and its regulators p35, p25, and p10 in XOR activation in response to hypoxia.

CDK5 was first discovered in the bovine brain over 20 years ago and was initially believed to have a function only in nerve tissue [24]. It is now well established that CDK5 is present in many different cell types and tissues and is an integral part of innumerable cellular processes including apoptosis, metabolism, proliferation, inflammation, and migration [25]. Previous investigators have demonstrated the importance of CDK5 in EC cell cycle regulation and resultant angiogenesis [26]. However, the role of CDK5 in XOR regulation has not been previously described. The present studies demonstrate that CDK5 and XOR are associated in a protein complex in EC (Fig 2). Neither this physical association nor the expression of CDK5 and XOR proteins were dynamically altered by hypoxic exposure. However, the enzymatic activity of both proteins is significantly increased in response to hypoxia.

In order to determine if CDK5 is a necessary upstream mediator of XOR hyperactivation under hypoxic conditions, we employed multiple complementary molecular and pharmacologic approaches, minimizing potential off-target or non-specific effects. Using both the CDK5 inhibitor olomoucine as well as ectopic expression of the dominant negative CDK5 enzyme (DNCDK5) [11], we demonstrated that the enzyme activity of CDK5 is required for hypoxia-induced hyperactivation of XOR in EC. Further, suppression of CDK5 using RNA interference (RNAi) with siRNA significantly attenuated hypoxia-induced XOR upregulation attesting to the specific role of CDK5 in hyperactivation. We also demonstrate a CDK5-dependent hyperactivation of XOR in the intact lung of animals challenged with hypoxia. Thus, we provide compelling evidence that CDK5 is obligatory for hypoxia-induced hyperactivation of XOR in EC.

CDK5 activity is controlled, in part, by binding to its regulatory subunit p35 and/or the p35-derived peptide p25, which contribute to CDK5 hyperactivation in certain disease states [17]. Recognizing that hypoxia increased CDK5 activity independent of changes in protein expression, we analyzed the expression and contribution of the known CDK5 activator, p35. Importantly, we demonstrate increased expression of p35 and p25 along with decreased levels of the inhibitory peptide, p10, in EC exposed to hypoxia (Fig 4) suggesting a potential mechanism of hypoxia-induced activation of CDK5. Since p25 and p10 are cleavage products of p35, we specifically targeted the parental regulator p35 with RNAi to address the contribution of p35, and thus p25, in hypoxia-induced hyperactivation of XOR. In the absence of p35, the hypoxia-induced activation of XOR is lost demonstrating the obligatory role of p35 and/or p25 in the regulation of XOR by hypoxia.

Another binding partner and potential activator of CDK5 is p39 which is homologous to p35 [27]. Unlike our observations with p35 and p25, we did not observe differences in p39 in EC exposed to hypoxia (data not shown) suggesting it is not dynamically altered by hypoxia in this cell type. While functional redundancy may exist between p35 and p39, or their cleavage products, our loss of function studies do not suggest that p39 is sufficient to activate XOR in the absence of p35, since hypoxia-induced activation of XOR was lost in p35-deficient EC. More formal investigation of a role for p39 or p29 in CDK5-dependent XOR regulation may be warranted in other contexts, but is beyond the scope of this paper. Collectively, our findings demonstrate both p35 and CDK5 are necessary for hypoxia-induced XOR hyperactivation.

Importantly, we also demonstrate that CDK5 is sufficient to increase XOR activity and that this CDK5-mediated hyperactivation is dependent on an intact CDK5 consensus sequence, specifically threonine 222. Consistent with our prior work *in vitro* [5], the hypoxia-induced hyperactivation of XOR occurs independent of changes in protein expression, implicating a post-translational mechanism. The sensitivity of XOR activity to alkaline phosphatase inactivation supports a critical role for the protein’s phosphorylation status in its regulation (Fig 1).

We identified a consensus target sequence for CDK5, a serine/threonine kinase, adjacent to the FAD binding domain within XOR. Here, we demonstrate that hypoxia-induced hyperactivation of XOR is associated with a significant increase in its immunoreactivity for phosphothreonine specific antibodies (Fig 2B). In addition, incubation of XOR with CDK5 in a cell free system is sufficient for phosphorylation and hyperactiviation of XOR (Figs 6 and 7) suggesting a role for threonine phosphorylation as a mechanism of post-translational regulation. To further define the specific contribution of the threonine within the putative CDK5 target site, we mutagenized the human XOR cDNA, replacing threonine (T) to alanine (A) at residue 222 (i.e. A222). The activities of the purified recombinant proteins were characterized in a cell-free reaction. The baseline activity of T222 XOR and A222 did not differ indicating that this threonine is not obligatory for basal XOR activity. When incubated with purified CDK5, the activity of T222 XOR significantly increased in an olomoucine-sensitive manner. Thus, CDK5 was sufficient to hyperactivate human XOR. In contrast, CDK5-dependent hyperactivation was completely lost in the A222 XOR mutant demonstrating that an intact CDK5 target site is required and that threonine 222 is necessary. These experiments confirm that CDK5-dependent activation of XOR requires threonine 222.

In conclusion, our findings demonstrate CDK5 is sufficient and necessary for hypoxia-induced XOR activation both *in vivo* and in pulmonary EC. Hypoxia increases expression of p35/p25 in EC and these CDK5 regulators are necessary for XOR hyperactivation. Finally, threonine 222 of XOR is the critical target site for CDK5-dependent activation of XOR. The link between XOR and CDK5, as well as, their importance in EC response to hypoxia are novel findings. Thus, we demonstrate a novel role for p35-CDK5 in the regulation of XOR.
